# Costunolide enhances doxorubicin-induced apoptosis in prostate cancer cells via activated mitogen-activated protein kinases and generation of reactive oxygen species

**DOI:** 10.18632/oncotarget.22592

**Published:** 2017-11-21

**Authors:** Jiasheng Chen, Binshen Chen, Zhihui Zou, Wei Li, Yiming Zhang, Jinlin Xie, Chunxiao Liu

**Affiliations:** ^1^ Department of Urology, Zhujiang Hospital of Southern Medical University, Guangzhou 510280, China; ^2^ Department of Urological Surgery, The Affiliated Luohu Hospital of Shenzhen University, Shenzhen University, Shenzhen 18000, China

**Keywords:** costunolide, apoptosis, prostate cancer, reactive oxygen species, MAPK

## Abstract

The management of castration-resistant prostate cancer (CRPC) is challenging, attributable to a lack of efficacious therapies. Chemotherapy is one of the most important treatments for CRPC. Doxorubicin has been extensively used in many different tumors and is often combined with other drugs to enhance effects and reduce toxicity. Costunolide is a natural sesquiterpene lactone with anti-cancer properties. In this study, we first demonstrated that the combination of costunolide and doxorubicin induced apoptosis significantly more than either drug alone in prostate cancer cell lines. Costunolide combined with doxorubicin induced mitochondria-mediated apoptosis through a loss of mitochondrial membrane potential and modulation of Bcl-2 family proteins. We found that this drug combination significantly increased the production of reactive oxygen species (ROS), as well as phosphorylation of c-jun N-terminal kinase (JNK) and p38 mitogen-activated protein kinases, which play upstream roles in mitochondria-mediated apoptosis. Further studies showed that N-acetyl cysteine blocked JNK and p38 phosphorylation, suggesting that ROS were upstream activators of JNK and p38. However, a JNK inhibitor, but not a p38 inhibitor, blocked the increase in ROS observed in cells treated with a combination of costunolide and doxorubicin, suggesting that ROS and JNK could activate each other. *In vivo*, inhibition of tumor growth and induction of apoptosis were greater in mice treated with the costunolide and doxorubicin combination than in mice treated with either drug alone, without an increase in toxicity. Therefore, we suggested that costunolide in combination with doxorubicin was a new potential chemotherapeutic strategy for treating prostate cancer.

## INTRODUCTION

Prostate cancer is commonly diagnosed in men living in western countries, and the incidence of prostate cancer in developing countries has been rapidly increasing in past decades [1, 2]. Although surgery and androgen ablation therapy are first-line treatments for prostate cancer, they often fail to be effective for advanced stage and castration-resistant prostate cancers (CRPCs) [3, 4]. Thus, chemotherapy remains a very important option for advanced-stage prostate cancer patients. In past years, drugs such as docetaxel have been first-line chemotherapeutics for CRPC [5]; however, prognoses remain very poor. Traditional chemotherapy is of limited use because of its low efficacy and high rate of side effects in normal tissues [6]. Hence, it is necessary to develop effective chemotherapeutics that increase cancer cell death at lower doses and with fewer side effects.

Doxorubicin (Dox) is the most frequently used anthracycline chemotherapeutic and it has previously been considered a highly effective mode of therapy for the treatment of prostate cancer [7]. However, its use has been limited, which is attributed to its high toxicity and side effects, including myelosuppression, gastrointestinal symptoms, cardiotoxicity, and chemoresistance in prostate cancer patients [8–10]. Combination therapy is a frequently used method in clinical practice to improve therapeutic effects and reduce the toxicity of anticancer drugs. Recent studies have shown that phytochemicals exhibiting anti-inflammatory and antioxidant properties can be used in combination with Dox to protect against cardiotoxicity [11]. Moreover, Dox has been used clinically in combination with other drugs such as 5-fluorouracil and mitomycin to effectively treat prostate cancers [7, 12].

Costunolide is a sesquiterpene lactone isolated from *Saussurea lappa* (costus roots) that exhibits anti-inflammatory and antioxidant properties and mediates apoptosis [13–15]. Previously, it has been shown that costunolide exerts anticancer activity through several signaling pathways, including activation of p53, activation of c-jun N-terminal kinase (JNK), inhibition of nuclear factor-κB and telomerase activity, and induction of apoptosis in several cancer lines, including a prostate cancer cell line [16, 17]. Costunolide has also been shown to increase the generation of reactive oxygen species (ROS), exert *in vivo* efficacy, and enhance cisplatin-induced cell death in ovarian cancer cells [18, 19]. These reports support costunolide as a drug candidate for further development.

In this study, we speculated that the combination of Dox and costunolide would result in enhancing anticancer activity in prostate cancer cell lines *in vitro* and *in vivo*. We used several methods to assess the anticancer effects of the drug combination in CRPCs. This study also provided evidence that the combination treatment induced apoptosis via signaling pathways.

## RESULTS

### Costunolide and Dox synergistically decreased survival of prostate cancer cells *in vitro*

**Figure 1:**
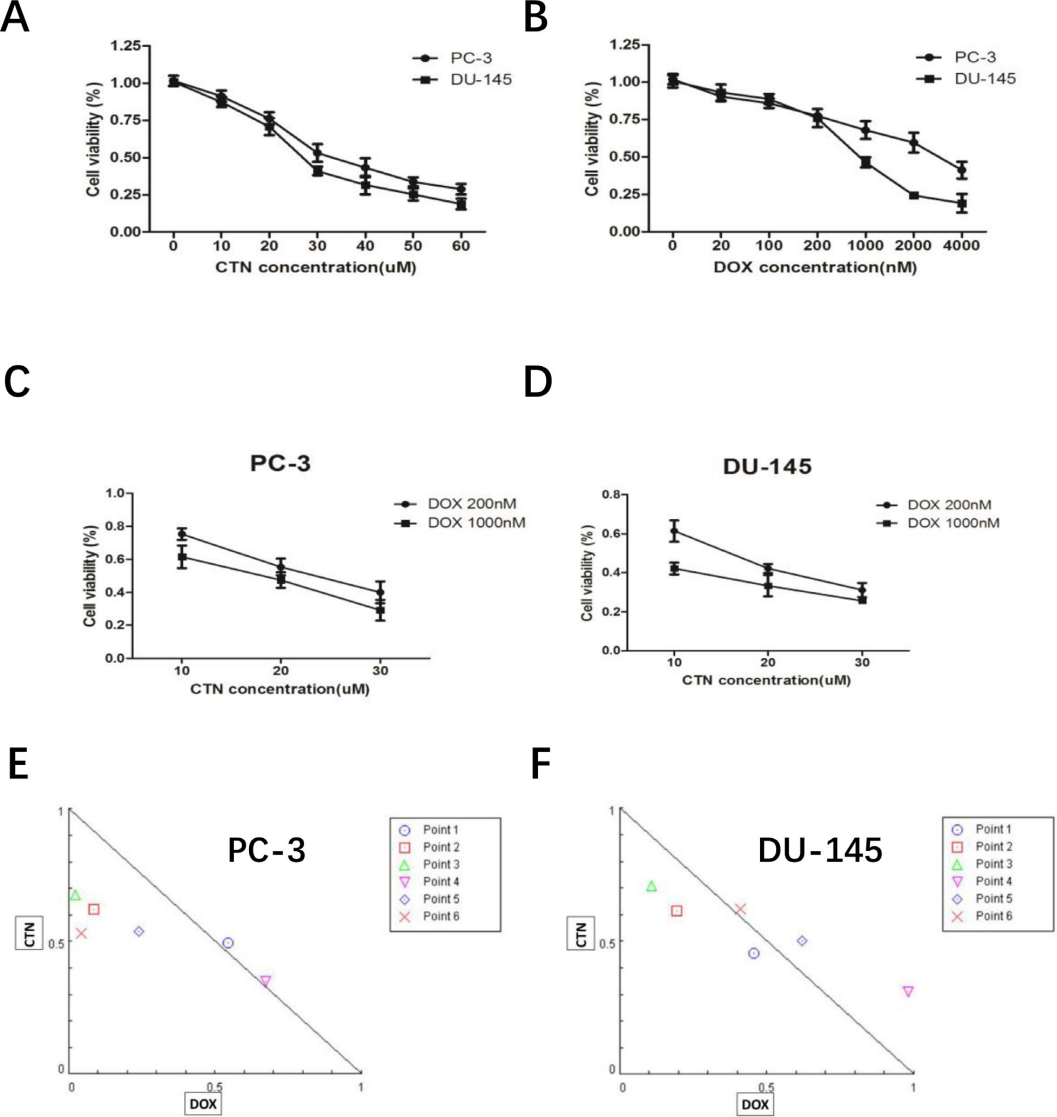
Treatment with costunolide or doxorubicin alone or combined two agents on cell proliferation of PC-3 and DU-145 (**A**) Inhibitory effect of costunolide treatment for 24 h. (**B**) Inhibitory effect of doxorubicin treatment for 24 h. Additionally, PC-3 (**C**) and DU-145 (**D**) cells were also exposed to different doses of costunolide that combine with 200 nM or 1000 nM doxorubicin. Combination index (CI) among the combinations of two drugs in PC-3 (**E**) and DU-145 (**F**) cells were calculated using CompuSyn software. If CI > 1, it denoted antagonism; if CI < 1, it denoted synergism. Here, points 1: CTN:10 μM + DOX:200 nM; 2: CTN:20 μM + DOX:200 nM; 3: CTN:30 μM + DOX:200 nM; 4: CTN:10 μM + DOX:1000 nM; 5: CTN:20 μM + DOX:1000 nM and 6: CTN:30 μM + DOX:1000 nM. Results were presented as the median of three independent experiments.

We investigated the effects of costunolide or Dox treatment on cell viability in the PC-3 and DU-145 prostate cancer cell lines. As shown in Figure 1A and 1B, both costunolide and Dox inhibited the growth of these cells in a concentration-dependent manner. The IC_50_ of costunolide in PC-3 cells was 34.92 μM, whereas its IC_50_ in DU-145 cells was 27.82 μM after exposure for 24 h. The IC_50_ for Dox was 3235.02 nM in PC-3 cells and 734.14 nM in DU-145 cells after exposure for 24 h. We investigated the potential synergy of costunolide and Dox to induce anti-cancer effects. We chose a low dose (200 nM) and a high dose (1000 nM) of Dox combined with different concentrations of costunolide (10, 20, and 30 μM) to treat PC-3 and DU-145 cells (Figure 1C and 1D). To analyze the combinational effects, we used CompuSyn software and the Chou-Talalay method to calculate the combination index (CI), which offers a quantitative definition for additive (CI = 1), synergistic (CI < 1), and antagonistic (CI > 1) effects of drug combinations. All the CI values were shown in the Supplementary Table 1. As shown in Figure 1E and 1F, the value of costunolide 20 μM + Dox 200 nM in both PC-3 (0.64 ± 0.086) and DU-145 (0.80 ± 0.035) cells were <1, which was suggestive of synergism. Therefore, we chose a concentration of 200 nM Dox and 20 μM costunolide for the treatment of both PC-3 and DU-145 cells in subsequent studies.

### Costunolide enhanced Dox-induced apoptosis in prostate cancer cells

**Figure 2:**
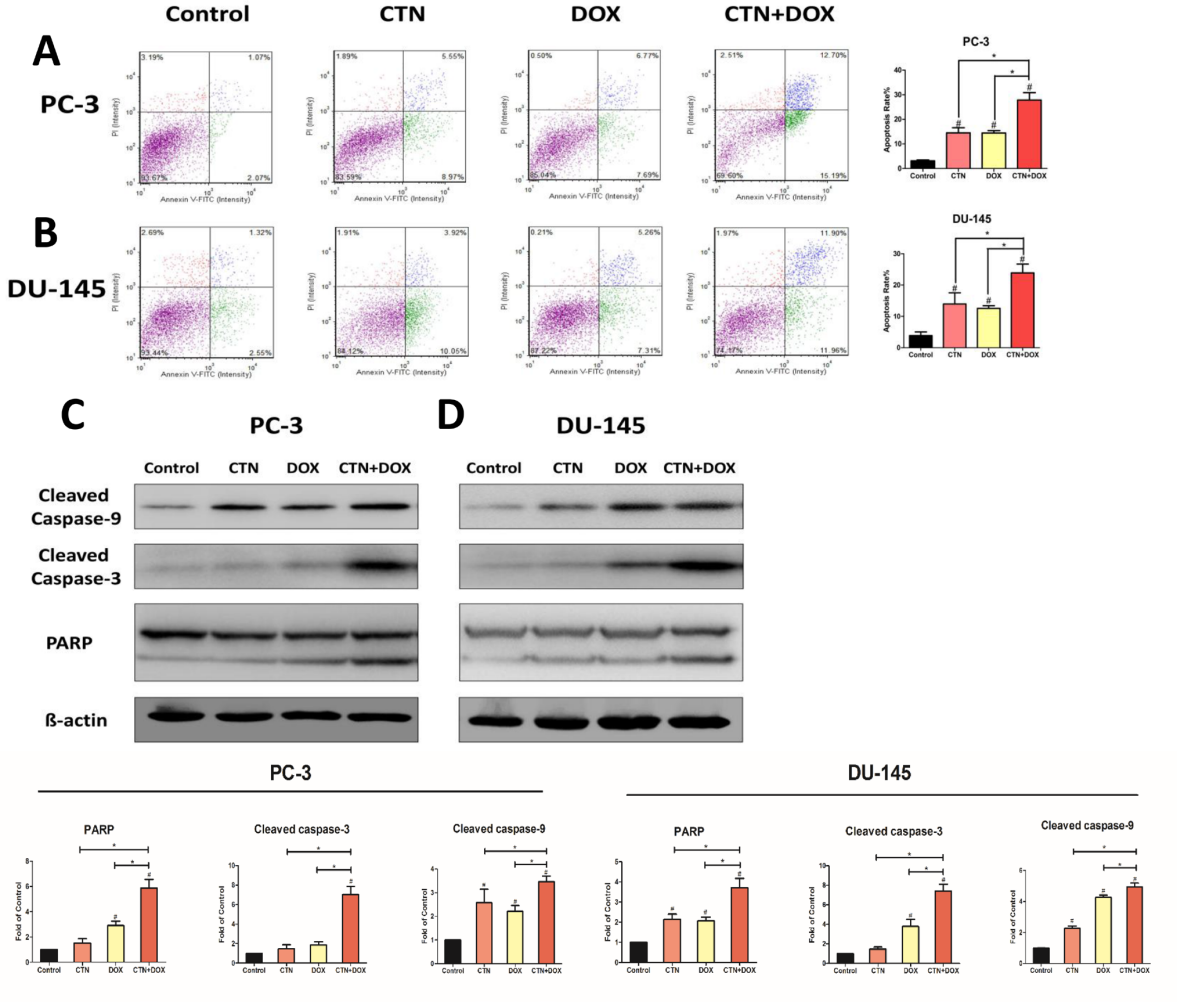
Costunolide enhanced doxorubicin to induce apoptosis in PC-3 and DU-145 cells PC-3 and DU-145 cells were treated with 20 μM costunolide, 200 nM doxorubicin, or both for 24 h. Costunolide enhanced doxorubicin to induce apoptosis in PC-3 (**A**) and DU-145 (**B**) cells detected by using annexin V-APC kit and PI staining. Bar charts showed quantitative data of average of 3 independent experiments in PC-3 and DU-145 cells. Western blots and quantitative analyses were performed to determine cleave caspase-9, cleave caspase-3 and PARP proteins expression in PC-3 (**C**) and DU-145 (**D**) cells after treatment. β-actin was used as a loading control. Results were presented as the mean ± SD of three independent experiments. ^*^p < 0.05; ^#^*p* < 0.05 compared with the control group.

Apoptosis is a major cause of cell growth inhibition; therefore, we hypothesized that decreased cell survival after treatment with the combination of agents was primarily attributable to increased apoptosis. Consequently, we used a cell viability counter (i.e., a cellometer) to analyze the induction of apoptosis in PC-3 and DU-145 cells treated with costunolide, Dox, or their combination for 24 h, and then stained with propidium iodide (PI)/annexin V. As shown in Figure 2A and 2B, the control group showed negligible apoptotic and necrotic cells (less than 5% in both DU-145 and PC-3 cells). An increase in cellular apoptosis was detected in the costunolide-only and Dox-only groups in both DU-145 and PC-3 cells, which agreed with previous reports showing that costunolide and Dox induced apoptosis of cancer cells. Costunolide induced cellular apoptosis at a rate of 14.52% in PC-3 cells and 13.97% in DU-145 cells. In contrast, Dox induced cellular apoptosis at a rate of 14.46% in PC-3 cells and 12.57% in DU-145 cells. Furthermore, costunolide synergistically enhanced Dox-induced apoptosis over that of either drug alone (27.89% in PC-3 cells and 23.86% in DU-145 cells). Significant differences were observed between all treatment groups and the control group (*P* < 0.05), with the combination group showing significant differences from the groups treated with either agent alone (*P* < 0.05). To analyze costunolide- and Dox-induced apoptosis, we detected caspase and poly (ADP-ribose) polymerase (PARP) activation via western blotting. As shown in Figure 2C and 2D, exposure to the combined agents led to higher levels of cleaved caspase-9, cleaved caspase-3, and cleaved PARP than that of costunolide or Dox alone in PC-3 and DU-145 cells. Taken together, these results demonstrated that costunolide greatly enhanced Dox-induced apoptosis in PC-3 and DU-145 cells.

### Costunolide enhanced Dox-induced mitochondrial dysfunction involving regulation of Bcl-2 family members in prostate cancer cells

**Figure 3:**
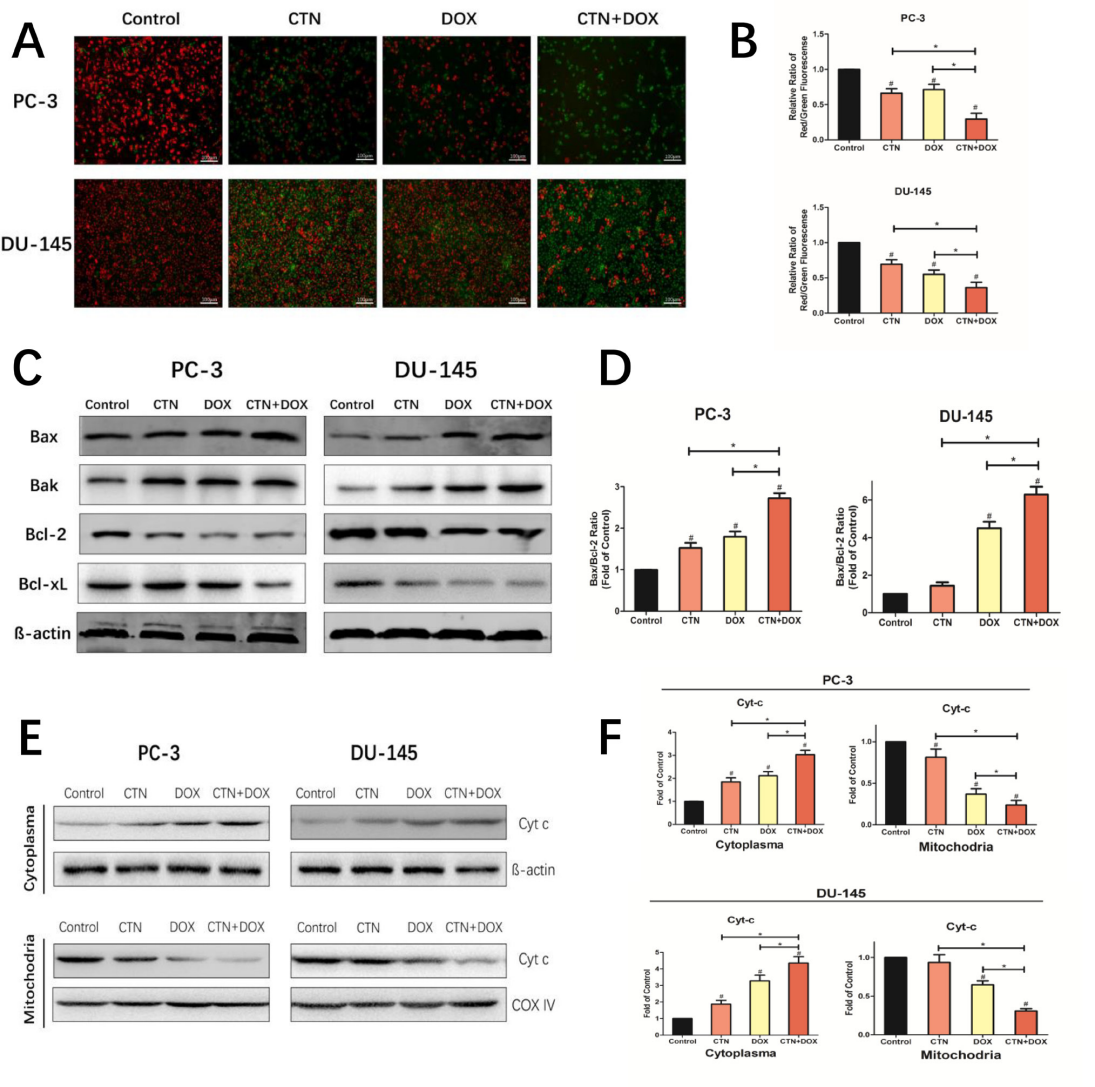
Costunolide enhanced doxorubicin to induce apoptosis through activating mitochondrial pathway in PC-3 and DU-145 cells PC-3 and DU-145 cells were treated with 20 μM costunolide, 200 nM doxorubicin, or both. (**A**) Costunolide enhanced doxorubicin to change of mitochondria membrane potential in the PC-3 and DU-145 cells by using the JC-1 kit. Scale bar was 100 μm. (**B**) Bar charts showed quantitative data of average of 3 independent experiments by flow cytometry. (**C**) Western blots were performed to determine expressions of Bax, Bak, Bcl-2, and Bcl-xL proteins in PC-3 and DU-145 cells after treatment. β-actin was used as a loading control. (**D**) The ratio of Bax/Bcl-2 were calculated from the bands corresponding to Bax and Bcl-2 that normalized to β-actin. Western blots (**E**) and quantitative analyses (**F**) were performed to determine cytosolic and mitochondrial fraction cytochrome C in PC-3 and DU-145 cells after treatment. COX IV and β-actin were used as loading controls for the mitochondrial fraction and the cytosolic fraction, respectively. Results were presented as the mean ± SD of three independent experiments. ^*^p < 0.05; ^#^*p* < 0.05 compared with the control group.

To determine whether mitochondrial dysfunction was involved in the apoptotic mechanism, we measured mitochondrial membrane potential (Δψm) in PC-3 and DU-145 cells after treatment with costunolide, Dox, or costunolide plus Dox (Figure 3A and 3B) using JC-1, a sensitive fluorescent probe. As shown in Figure 3A and 3B, the fluorescence was transformed from red (high Δψm, aggregated JC-1) to green (low Δψm, monomeric JC-1) in the treated groups. Costunolide decreased Δψm at a rate of 33.65% in PC-3 cells and 31.64% in DU-145 cells. In contrast, Dox decreased Δψm at a rate of 28.54% in PC-3 cells and 45.15% in DU-145 cells. Furthermore, costunolide synergistically enhanced Dox-decreased Δψm over that of either drug alone (70.43% in PC-3 cells and 63.77% in DU-145 cells). Significant differences were observed between all treatment groups and the control group (*P* < 0.05), with the combination group showing significant differences from the groups treated with either agent alone (*P* < 0.05).

The imbalance in expression of proapoptotic and antiapoptotic Bcl-2 family members determines the fate of cells. To assess whether the mitochondrial pathway was involved in costunolide- and Dox-induced apoptosis, we detected changes in the expression of proapoptotic Bcl-2 members (Bax and Bak), antiapoptotic Bcl-2 members (Bcl-2 and Bcl-xL) via western blotting. As shown in Figure 3C, the expressions of Bcl-2 and Bcl-xL were decreased, whereas the levels of Bax and Bak were increased in cells treated with costunolide combined with Dox. We found that treatment with costunolide combined with Dox synergistically increased the ratio of Bax/Bcl-2 compared to that of treatment with either drug alone in PC-3 and DU-145 cells (*P* < 0.05, Figure 3D). Next, we detected changes in the expression of cytochrome *C* in cytosolic and mitochondrial fractions. As shown in Figure 3E and 3F, mitochondrial cytochrome *C* expressions were decreased, whereas the levels of cytosolic cytochrome *C* were increased in PC-3 and DU-145 cells treated with costunolide combined with Dox, compared to that of costunolide or Dox alone (*P* < 0.05). Thus, these data demonstrated that treatment with costunolide plus Dox synergistically increased mitochondrial dysfunction, which favors the induction of apoptosis.

### Costunolide combined with Dox increased ROS levels in prostate cancer cells

**Figure 4:**
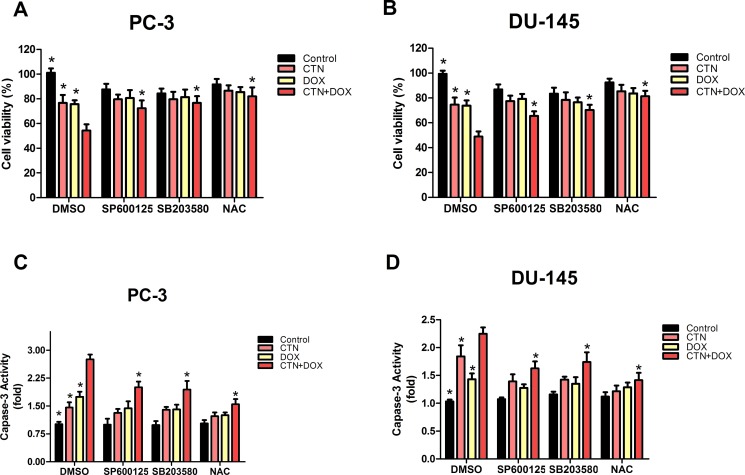
Costunolide enhanced the cytotoxic effect of doxorubicin via activated the p38, JNK pathways and ROS in PC-3 and DU-145 cells PC-3 and DU-145 cells were treated with 20 μM costunolide, 200 nM doxorubicin, or both in the absence or presence ROS scavenger (NAC, 5 mM), JNK inhibitor (SP600125, 10 μM) and P38 inhibitor (SB203580. 10 μM). Using CCK-8 assay to detect the cell viability in PC-3 (**A**) and DU-145 (**B**) cells, we found that percentage of survived cells in the CTN + DOX group statistically significant decrease (^*^p < 0.05, compared with CTN + DOX group), but this observation was reversed significantly when we added the JNK inhibitor (SP600125), P38 inhibitor (SB203580), ROS scavenger (NAC) to the CTN + DOX group (^*^p < 0.05, compared with CTN + DOX group). Using the caspase-3 activity assay kit to detect the apoptosis in PC-3 (**C**) and DU-145 (**D**) cells, we found that caspase-3 activity increased significantly in the CTN + DOX group compared with either agent alone group (^*^p < 0.05, compared with CTN + DOX group), but this observation was reversed significantly when we added the JNK inhibitor (SP600125), P38 inhibitor (SB203580), ROS scavenger (NAC) to the CTN + DOX. (^*^p < 0.05, compared with CTN + DOX group). Data presented as mean ± SD were representative of three independent experiments.

**Figure 5:**
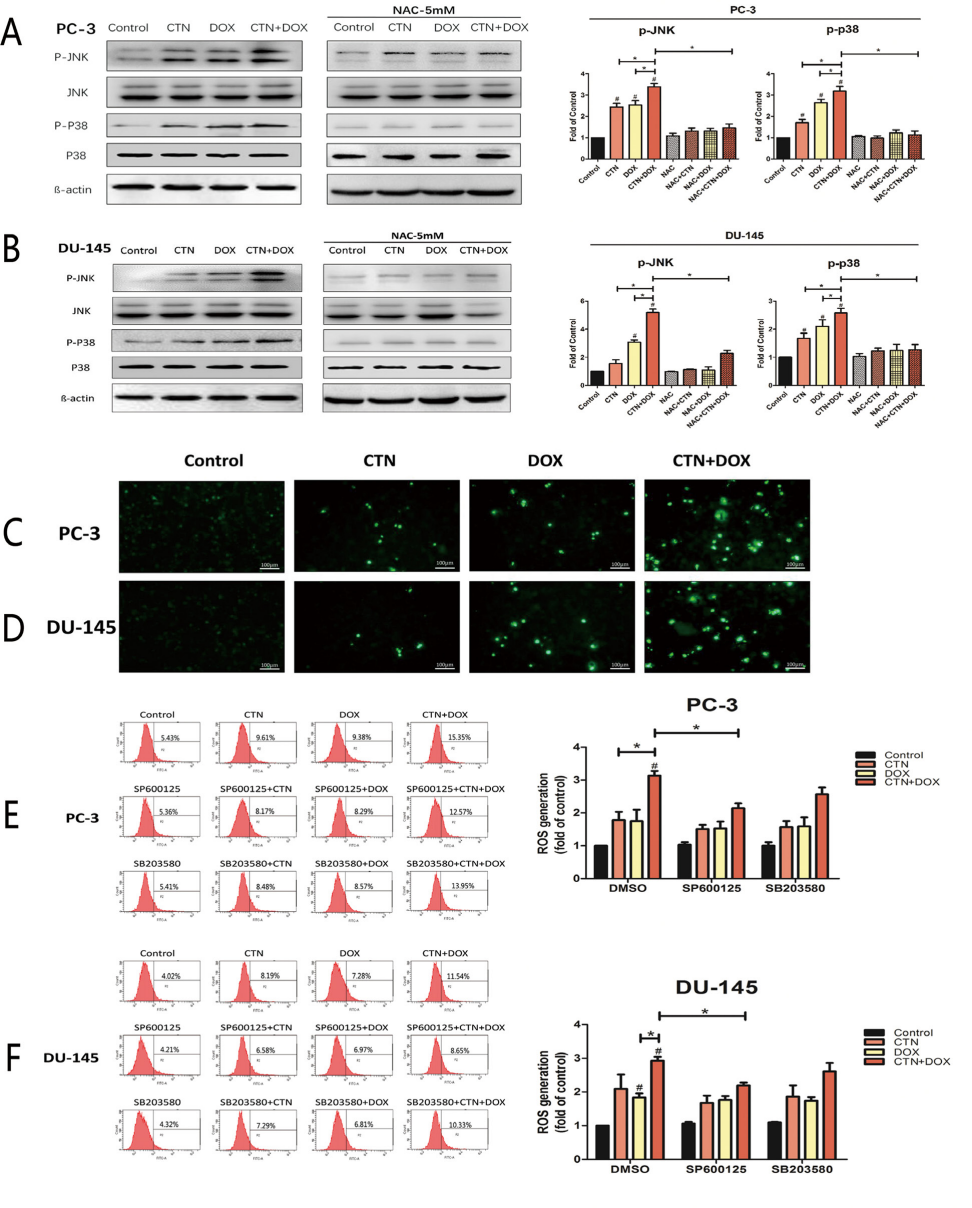
Involvement of p38 and JNK in ROS induction by the combination of costunolide and doxorubicin PC-3 (**A**) and DU-145 (**B**) were treated with 20 μM costunolide, 200 nM doxorubicin, or both in the absence or presence ROS scavenger (NAC, 5 mM), then the western blots and quantitative analyses were performed to determine the p-p38, p-JNK proteins expression in PC-3 and DU-145 cells after treatment. β-actin was used as a loading control. PC-3 (**C**) and DU-145 (**D**) were treated with 20 μM costunolide, 200 nM doxorubicin, or both, and then ROS assay was performed, and captured under a fluorescence microscope. Scale bar was 100 μm. PC-3 (**E)** and DU-145 (**F**) were treated with 20 μM costunolide, 200 nM doxorubicin, or both in the absence or presence JNK inhibitor (SP600125, 10 μM) and P38 inhibitor (SB203580, 10 μM) respectively, then ROS production was measured using DCFH-DA by flow cytometry. Bar charts showed quantitative data of average of 3 independent experiments. ^*^p < 0.05; ^#^*p* < 0.05 compared with the control group.

To determine whether the induction of apoptosis resulting from treatment with the combination of costunolide and Dox in PC-3 and DU-145 cells were mediated by elevated ROS, we included N-acetyl cysteine (NAC, 5 mM), a ROS scavenger, and investigated alterations in cytotoxic effects using the cell counting kit-8 (CCK-8) assay and caspase-3 activity assay. PC-3 and DU-145 cells were pretreated with NAC for 2 h, treated with costunolide, Dox, or their combination for 24 h, and then were analyzed. As shown in Figure 4, cell proliferation was significantly increased and caspase-3 activation was inhibited in cells treated with the combination of costunolide and Dox in the presence of NAC (*P* < 0.05). To analyze the role of ROS in costunolide-and Dox-induced apoptosis, we measured ROS generation using fluorescence microscopy (Figure 5C and 5D) and flow cytometry (Figure 5E and 5F). The fold changes in ROS levels between the control cells and the cells treated with costunolide, Dox, or costunolide plus Dox for 24 h were 1.78, 1.74, and 2.83, respectively, in PC-3 cells, and 2.09, 1.84, and 2.92, respectively, in DU-145 cells. The results for the combination treatment were significantly increased in PC-3 and DU-145 cells. (*P* < 0.05). These data suggested that the combination of costunolide- and Dox increased ROS generation to induce apoptosis more than that of either drug alone.

### Costunolide and Dox activated p38 and JNK to induce apoptosis in prostate cancer cells

To better understand the mechanisms underlying costunolide- and Dox-induced cellular apoptosis in PC-3 and DU-145 cells, we investigated the role of MAPK signaling pathways. We found that treatment with costunolide combined with Dox synergistically increased phosphorylation of p38 and JNK proteins compared to that of treatment with either drug alone in PC-3 and DU-145 cells (Figure 5A and 5B). To confirm involvement of the MAPKs, we used a p38 inhibitor (SB203580, 10 μM) and JNK inhibitor (SP600125, 10 μM) to investigate alterations in the cytotoxic effects using the CCK-8 and caspase-3 activity assays. PC-3 and DU-145 cells were pretreated with SB203580 or SP600125 for 2 h, treated with costunolide, Dox, or their combination for 24 h, and then were analyzed. As shown in Figure 4, SB203580 or SP600125 significantly attenuated the decreased cell proliferation and increased caspase-3 activation observed with the combined treatment of Dox and costunolide (*P* < 0.05). Taken together, these data indicated that the synergistic effects of costunolide and Dox in PC-3 and DU-145 cells might be dependent on activating both p38 and JNK pathways.

### Role of p38 and JNK pathways in ROS induced by costunolide combined with Dox

Costunolide enhanced Dox-induced ROS generation and increased the phosphorylation of p38 and JNK, suggesting a role for upstream activators of the mitochondria-mediated apoptosis pathway. Therefore, we used NAC (5 mM), along with SB203580 (10 μM) and SP600125 (10 μM), to determine the interrelationship. As shown in Figure 5E and 5F, pretreatment with SP600125 for 2 h followed by treatment with costunolide, Dox, or costunolide plus Dox for an additional 24 h resulted in less ROS generation than that in the combination treatment (*P* < 0.05); however, this phenomenon did not occur in cells treated with SB203580. Furthermore, we pretreated cells with NAC for 2 h followed by treatment with costunolide, Dox, or costunolide plus Dox for an additional 24 h and then detected p38 and JNK phosphorylation by western blot. As shown in Figure 5A and 5B, p38 and JNK phosphorylation were significantly inhibited in the NAC-pretreated group, suggesting that p38 and JNK phosphorylation were activated by the generation of ROS.

### Costunolide enhanced Dox-induced apoptosis *in vivo*

**Figure 6:**
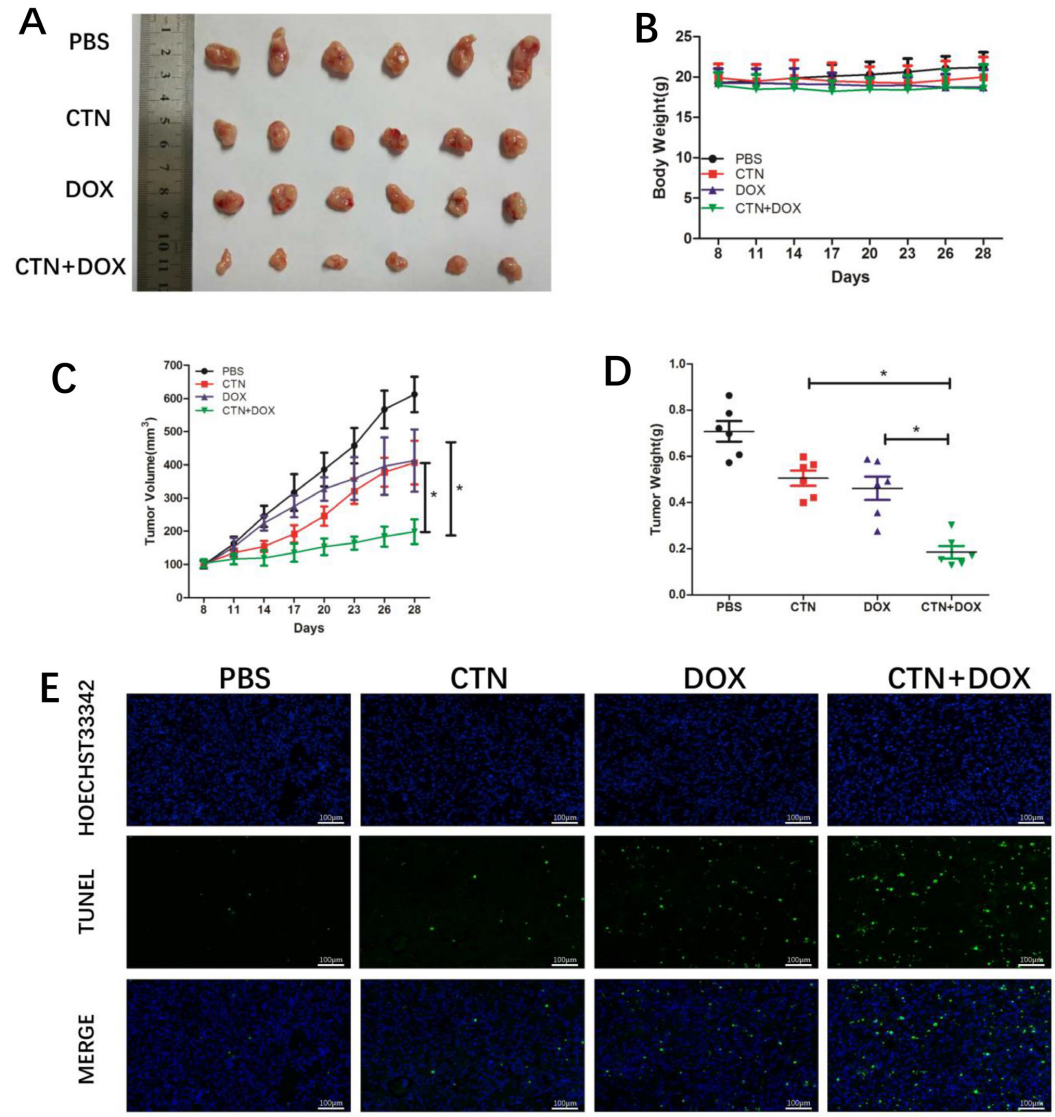
Costunolide enhanced doxorubicin to inhibit PC-3 tumor growth in athymic nude mice via induction of apoptosis Nude mice inoculated with PC3 cells were treated with costunolide, doxorubicin alone or combined two agents. (**A**) Tumors were photographed at the end of treatment (Day 21). (**B**) Mouse body weights were monitored every three days for three weeks till the end of treatment (Day 21). (**C**) Tumor growth curves based on tumor volumes measured every three days and (**D**) weights recorded at the end of treatment (Day 21) (^*^p < 0.05). (**E**) Tumor tissues were examined by TUNEL assay (100×). Scale bar was 100 μm.

Costunolide combined with Dox effectively induced apoptosis in PC-3 and DU-145 cells; therefore, we extended our study to determine whether these events occur *in vivo* using a xenograft mouse model. Nude mice xenografts were randomly divided into four groups, including a control group, costunolide group (5 mg/kg, every 3 days), Dox group (1 mg/kg, every 3 days), and a costunolide and Dox combination group. After treatment for three weeks, tumor growth was slower in animals treated with costunolide plus Dox than in animals treated with either drug alone (Figure 6A). Furthermore, there were no differences observed in the body weights of the mice among the groups (Figure 6B). In contrast, tumor volume and tumor weight were significantly less in mice treated with the combination of costunolide and Dox than the other treatment groups (*P* < 0.05) (Figure 6C and 6D).

**Figure 7:**
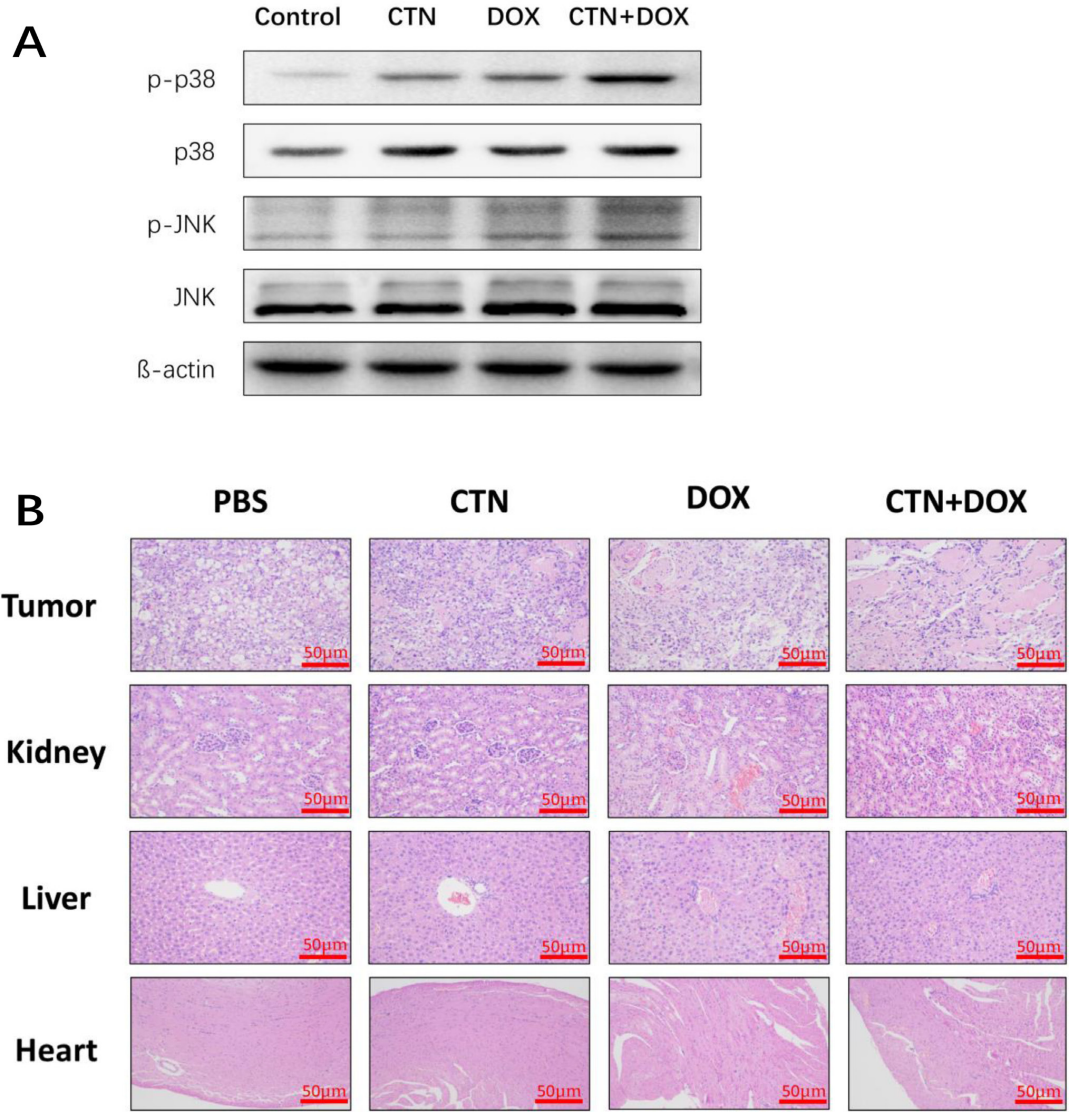
Western blot analyses of tumor tissues and HE staining of the tissue slices from xenograft tumors and organs At the end of experiment, (**A**) the western blots were performed to determine the expressions of p-p38, p-JNK proteins in tumor tissue. β-actin was used as a loading control. (**B**) The nude mice were sacrificed, and then tumor, liver, kidney and cardiac tissue were excised and re-examined by H&E staining (50×). Scale bar was 50 μm. The treatment group had no abnormal histological changes of liver, kidney and cardiac tissues in nude mice compared to the control group.

We next investigated the antitumor effects and safety profiles of costunolide, Dox, and costunolide plus Dox. We performed terminal deoxynucleotidyl transferase dUTP nick end labeling (TUNEL) assays to detect DNA fragmentation resulting from apoptosis. Green fluorescence was observed more in tumor tissues from costunolide plus Dox-treated mice than in tumor tissues from other treatment groups, which was indicative of apoptosis in the former (Figure 6E). Hematoxylin and eosin (H&E) staining of tumors showed more obvious necrotic areas in the combination agent group than those in the other treatment groups. No pathological changes were observed in all the heart, liver and kidney sections. These results showed that the combination of drugs resulted in no significant toxicity in the mice (Figure 7B). Western blot analysis showed that costunolide plus Dox treatment group resulted synergistically increased phosphorylation of p38 and JNK proteins compared to that of treatment with either drug alone (Figure 7A), which was consistent with our findings in cell culture.

## DISCUSSION

Chemotherapy has been one of the most important treatment options for CRPC; however, side effects have limited the scope of drug usage. Clinically, a commonly used measure to prevent Dox-induced side effects is to limit the dosage of Dox, but this could result in reduced Dox efficacy with an associated increase in cancer mortality. Therefore, it is necessary to find novel therapeutic strategies to enhance the anticancer effects and reduce the side effects resulting from chemotherapy [7, 12]. For this purpose, the use of natural compounds for combinational therapies is a popular approach to combat the negative aspects associated with current chemotherapeutic agents [20].

Previous studies have shown that costunolide has antioxidant or pro-oxidant effects depending on its concentration or the target cells, and it can induce cancer cell apoptosis by increasing ROS production. In the present study, we found that costunolide enhanced the anticancer effects of Dox *in vitro* and *in vivo*. Costunolide and Dox markedly decreased the viability of prostate cancer cells (PC-3 and DU-145) in a concentration-dependent manner, as was previously shown (Figure 1). Of note, our data showed that the combination of 20 μM CTN and 200 nM DOX results in decreased cell survival of PC-3 and DU-145 cells, with a CI value < 1 (Figure 1E and 1F), suggesting that the two agents have a synergistic effect. Thus, we chose the two concentrations of the CTN and DOX as the following study. As we know, apoptosis is the primary mechanism of cell death; many chemotherapeutic agents are known to induce apoptosis to prevent tumor promotion and progression [21, 22]. Our results showed that the combination of costunolide and Dox significantly increased apoptosis in PC-3 and DU-145 cells, as demonstrated using viable cell counting and western blotting (Figure 2). Moreover, the combination therapy was more effective at inhibiting tumor growth than either drug alone in the xenograft model, and the combination did not induce histopathological changes in the cardiac, hepatic and renal tissues (Figure 7). Histological analysis revealed more obvious necrotic areas at the tumor in the combination group compared to that in the other groups, and the TUNEL assay revealed that Dox induced tumor cell apoptosis, which was enhanced by costunolide (Figure 6E). Collectively, these results demonstrated a synergistic effect between costunolide and Dox.

Mitochondrial dysfunction is an important classical pathway for inducing apoptosis that depends on the release of cytochrome C from the mitochondria to the cytosol, resulting in activation of caspase-9 [23–25]. Activation of the mitochondria-mediated apoptotic pathway is a well-characterized mechanism of anticancer drugs. In the study, we found that costunolide enhances Dox-induced apoptosis by activating the mitochondria-mediated apoptotic pathway. The Bcl-2 family of proteins can be subdivided into antiapoptotic and pro-apoptotic members, which have been shown to be central regulators of Δψm [26]. And the balance between the levels of Bcl-2 and Bax is critical in determining cell apoptosis. The results demonstrated that the combination treatment significantly increased the ratio of Bax/Bcl-2, and promoted the redistribution of cytochrome C. Furthermore, costunolide enhanced Dox-induced loss of Δψm in PC-3 and DU-145 cells (Figure 3), suggesting that the two agents had synergistic effects toward mitochondrial dysfunction. These results further indicated that the combination of costunolide and Dox caused PC-3 and DU-145 cellular apoptosis by inducing mitochondrial dysfunction.

ROS are likely indispensable for signal-transduction pathways that regulate cell growth and redox status [27]. However, overproduction of ROS can damage lipids, proteins, and DNA. Mitochondria is a primary source of ROS generation, which is highly integrated during apoptosis [28, 29]. Excessive ROS production induces mitochondrial permeability transition (MPT), which leads to loss of Δψm and activates mitochondrial apoptosis. Therefore, we examined the possibility that ROS induce apoptosis. Costunolide enhanced Dox-induced anticancer effects by increasing ROS levels in cancer cells, as shown by dichloro-dihydro-fluorescein diacetate (DCFH-DA) fluorescent labeling and flow cytometry (Figure 5C–5F). Furthermore, our results showed that NAC inhibited costunolide plus Dox induced cell growth and caspase-3 activity in PC-3 and DU-145 cells (Figure 4), confirming that ROS play an upstream role in the apoptosis pathway.

MAPKs are a widely conserved family of serine/threonine protein kinases involved in many cellular activities such as proliferation, differentiation, motility, and death [30]. Evidence suggests that MAPK signaling pathways play important roles in the action of some chemotherapeutic drugs and the regulation of apoptosis. Extracellular signal-regulated kinase (ERK), JNK, and p38 are important mediators in MAPK signaling pathways. Generally, JNK and p38 activation enhances cell death, while ERK activation induces cell proliferation [31]. Dox increases the phosphorylation of p38 and JNK proteins to induce cellular apoptosis in many cancer cell lines, including prostate cancer cells. Previously, costunolide was shown to induce apoptosis through the JNK signaling pathway [17, 32–34]. We detected the phosphorylation of p38 MAPK and JNK proteins in PC-3 and DU-145 cells and found the expressions were dose dependence, so it meant that the CTN also could active p38 MAPK and JNK in prostate cancer cells (Supplementary Figure 1). Furthermore, we found that the combination of costunolide and Dox induced p38 and JNK phosphorylation more than either agent alone *in vitro* and *vivo*. In addition, inhibition of p38 and JNK phosphorylation by SB203580 and SP600125, respectively, attenuated costunolide plus Dox-induced inhibition of PC-3 and DU-145 cell growth and activation of caspase-3 (Figure 4), suggesting that p38 and JNK were involved in the apoptosis induced by costunolide plus Dox.

Numerous studies have reported that proapoptotic p38 and JNK MAPK are activated by ROS [35–37]. In the p38 and JNK signaling pathway, ROS induce the activation of MAPK/ ERK kinase 1 (MEKK1), mixed-lineage protein kinase 3 (MLK3), and apoptosis signal-regulating kinase 1 (ASK1), which are MAPK kinase kinases (MAPKKK); the MAPKKK active MAPK kinases and then activate p38 and JNKs [38, 39]. Our results showed that NAC inhibited the phosphorylation p38 and JNK (Figure 5A and 5B), suggesting that ROS were the common upstream regulators of both MAPK. NADPH oxidases (NOX) are widely expressed in tissues, and their biological functions include generation of ROS. Recent studies have shown that NOX are overexpressed in several types of cancer, including prostate cancer, and they can be up-regulated by the JNK pathway [40, 41]. We used SP600125 and SB203580 to determine the relationship between ROS and the MAPK pathways and found that SP600125 inhibited the production of ROS in the combination treatment group; however, this phenomenon was not observed in the SB203580-treated groups (Figure 5E and 5F). These data suggested that the generation of ROS was activated by JNK, but not p38, phosphorylation. Therefore, the JNK pathway and ROS may activate each other in the presence of costunolide and Dox combination treatments, and these activation mechanisms were not observed in the presence of either agent alone. These potential mechanisms were similar to those of the signaling mechanisms of JNK-NADPH oxidase-ROS, which have been described in hepatic carcinoma cells [42, 43]. These data provided a potential mechanism for costunolide enhancement of Dox-induced cancer cell apoptosis.

In conclusion, our results showed that costunolide enhanced Dox-induced apoptosis in the PC-3 and DU-145 cell lines through a mitochondria-mediated apoptotic pathway by facilitating crosstalk of p38/JNK pathways and ROS generation. And the combination therapy was more effective and lower toxicity at inhibiting tumor growth than either drug alone *in vivo*. These findings suggest a potential application for costunolide in clinical combined therapies against prostate cancer. However, this study has some limitations. For instance, the mechanism underlying the interactions between mitochondrial ROS and JNK is unclear. As such, more research needs to be conducted in the future.

## MATERIALS AND METHODS

### Chemicals

Roswell Park Memorial Institute medium (RPMI)-1640, Dulbecco's modified Eagle's medium (DMEM), and fetal bovine serum (FBS) were purchased from GIBCO Invitrogen (Carlsbad, CA, USA). Costunolide, Dox, SP600125 (JNK inhibitor), SB203580 (p38 MAPK inhibitor), phenylmethylsulfonyl fluoride (PMSF), NAC, and all other chemical compounds were obtained from Sigma-Aldrich (St. Louis, MO, USA). The antibodies against phosphorylated p38 (Thr180/Tyr182), phosphorylated JNK (Thr183/Tyr185), total p38, total JNK, Bak, Bax, Bcl-2, Bcl-xL, cytochrome c, cleaved caspase-9, cleaved caspase-3, PARP, and β-actin were purchased from Cell Signaling Technology (Danvers, MA, USA).

### Cell culture

The human PC-3 and DU-145 prostate cancer cell lines were purchased from ATCC (Manassas, VA, USA) and were cultured in RPMI-1640 medium supplemented with 10% FBS and 1% penicillin-streptomycin at 37°C in a 5% CO_2_ atmosphere.

### Proliferation assay

Cell viability was evaluated using the CCK-8 assay (Beyotime Biotechnology, Jiangsu, China). The cells were seeded overnight in 96-well plates at a density of 5,000 cells per well, and then treated with costunolide, Dox, or costunolide plus Dox for 24 h. After treatment with the indicated drugs, 10 μL CCK-8 was added to each well. The plates were incubated for 2 h at 37°C and then vigorously shaken; absorbance was measured at 540 nm. Drug interactions were analyzed using the median effect principle (Talalay-Chou method) [44]. A CI was used to find the synergistic combination dose. The CI was calculated using CompuSyn software, where a CI = 1 was indicative of an additive effect, a CI < 1 was indicative of a synergistic effect, and a CI > 1 was indicative of an antagonistic effect.

### Apoptosis assay

Induction of apoptosis was detected with a PI/annexin V staining kit (Biolegend, San Diego, CA, USA). Briefly, 3 × 10^5^ cells/well were seeded into 6-well plates and incubated overnight. Treatments were then added, and the cells were washed thrice with PBS and stained with Annexin V (1 μl) and PI (0.5 μl, 10 mg/ml) for 20 min in the dark. The samples were immediately measured using a cellometer (Nexcelom, San Francisco, CA, USA), and the results were analyzed using FCS5 express software. Total apoptotic cells (early apoptosis + late apoptosis) are expressed as a percentage of the total number of stained cells. All experiments were performed in triplicate to assess for consistency of response.

### ROS assay

ROS were detected using DCFH-DA (Beyotime Biotechnology, Jiangsu, China) and measured using flow cytometry (BD, Biosciences, USA) and fluorescence microscopy (Zeiss, Jena, Germany). Briefly, 2 × 10^5^ PC-3 or DU145 cells were prestained with the ROS working solution for 1 h and then treated with DCFH-DA for 20 min. All experiments were performed in triplicate.

### Mitochondrial membrane potential determination

A JC-1 kit (Beyotime Biotechnology, Jiangsu, China) was used to detect changes in Δψm. Briefly, 3 × 10^5^ cells/well were seeded into 6-well plates and incubated overnight. Treatments were then added, and the cells were washed thrice with PBS and stained with JC-1 (200 μM) for 1 h in the dark. Changes in Δψm were measured using flow cytometry (BD, Biosciences, USA) and fluorescence microscopy (Zeiss, Jena, Germany). All experiments were performed in triplicate to assess for consistency of response.

### Caspase-3 activity assay

Caspase-3 activity was detected using a caspase-3 activity kit (Beyotime Biotechnology, Jiangsu, China), according to the manufacturer's instructions. Cellular extracts were incubated in 96-well plates for 2 h at 37°C with caspase-3 substrate (Ac-DEVD-pNA). Samples were measured with an enzyme-linked immunosorbent assay (ELISA) plate reader at a wavelength of 405 nm. Caspase-3 activity is expressed as the percentage of enzyme activity compared to that of control. All experiments were performed in triplicate.

### Western blot

Total proteins were extracted from cells and tisues using radioimmunoprecipitation assay (RIPA) buffer (Thermo Fisher Scientific, MA, USA) and quantified with the bicinchoninic acid (BCA) assay kit (Thermo Fisher Scientific). Cytochrome c release from mitochondria was evaluated by western blot analysis of cytosolic protein samples. Cytosolic and mitochondrial protein fractions were prepared using the cell mitochondria isolation kit (Beyotime, JiangSu, China). Equal amounts (30–50 μg) of total proteins were loaded and separated by 10–12% sodium dodecyl sulfate polyacrylamide gel electrophoresis (SDS-PAGE), transferred to a polyvinylidene difluoride (PVDF) membrane, blocked in 5% non-fat milk, and incubated with primary antibodies overnight at 4°C. Subsequently, the membranes were incubated with the appropriate secondary antibodies for 1 h at 25°C before development. The bands were developed and quantified using an Alpha Innotech imaging system (San Leandro, CA, USA). Each experiment was repeated three times to assess for consistency of results. β-actin was used as the loading control.

### *In vivo* studies

BALB/c nude mice (24 male) were purchased from the Animal Center of Southern Medical University and all animal experiments were approved by the Ethics Committee of Southern Medical University. PC-3 cells (3 × 10^6^) were injected subcutaneously into the armpit of each mouse. The body weight of each mouse was recorded daily and tumor growth was monitored every three days. When the average tumor volume reached 100 mm^3^, the tumor-bearing mice were randomly divided into four groups and were treated for 3 weeks with vector (PBS), 5 mg/kg/3 day costunolide, 1 mg/kg/3 day Dox, or the combination of costunolide and Dox. Tumor growth and body weight of the mice were monitored daily. Tumor volume was calculated from the formula V = 1/2 (A × B^2^), where A was the longest diameter and B was the perpendicular diameter as measured with calipers.

### TUNEL assay

A TUNEL assay was performed to detect apoptotic cells, according to the manufacturer's instructions (TUNEL Apoptosis Detection Kit, GenScript, Piscataway, NJ, USA). All sections were assessed under a microscope (Nikon, Japan). For each group, the number of apoptotic cells and the total number of cells in five random fields (magnification, ×100) were photographed.

### Histological analysis

After the mice were euthanized, heart, livers, kidneys and tumors were collected, fixed in 4% paraformaldehyde for 24 h, and subsequently embedded in paraffin. Tissue sections (5 μm) were stained with H&E.

### Statistical analysis

All data are presented as means ± SD. Data were analyzed by analysis of variance (ANOVA) using SPSS ver. 17.0 (Armonk, NY, USA). Tukey's honestly significant difference (HSD) test was used for post-hoc comparisons. *P* < 0.05 was considered statistically significant.

## SUPPLEMENTARY MATERIALS FIGURES AND TABLES


