# Supplementary material for: Costunolide enhances doxorubicin-induced apoptosis in prostate cancer cells via activated mitogen-activated protein kinases and generation of reactive oxygen species
Source: Oncotarget. 2017 Nov 21;8(64):107701–15. doi: 10.18632/oncotarget.22592 (PMC5746101; doi:10.18632/oncotarget.22592)
Supplement: Supplementary file 1 [file oncotarget-08-107701-s001.pdf]

Costunolide enhances doxorubicin-induced apoptosis in prostate cancer cells via activated mitogen-activated protein kinases and generation of reactive oxygen species

SUPPLEMENTARY MATERIALS

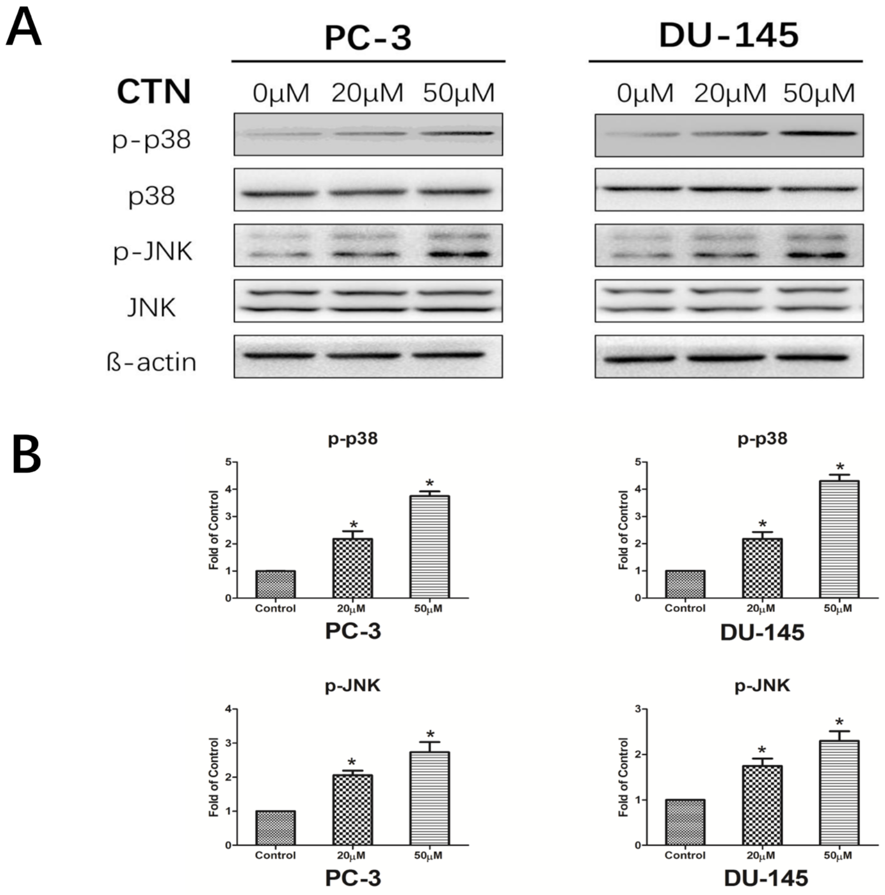

Supplementary Figure 1: Costunolide activated the p38, JNK pathways by dose dependence in PC-3 and DU-145 cells. PC-3 and DU-145 cells were treated with 20 µM, 50 µM costunolide for 24 h. Then (**A**) the western blots and (**B**) quantitative analyses were performed to determine the expressions of p-p38, p-JNK proteins in PC-3 and DU-145 cells after treatment. β-actin was used as a loading control. Data presented as mean ± SD were representative of three independent experiments ($^{*}P < 0.05$).

Supplementary Table 1: Combination index (CI) of CTN and DOX combination in PC-3 and DU-145 treated cells

Prostate cancer cell lines	Combination dose	Cell viability	Combination index (CI)	Effects
**PC-3**	CTN 10 µM + DOX 200 nM	0.75 ± 0.034	1.18 ± 0.304	——
	CTN 20 µM + DOX 200 nM	0.51 ± 0.05	0.64 ± 0.086	Synergistic
	CTN 30 µM + DOX 200 nM	0.39 ± 0.067	0.71 ± 0.123	Synergistic
	CTN 10 µM + DOX 1000 nM	0.61 ± 0.069	1.27 ± 0.618	——
	CTN 20 µM + DOX 1000 nM	0.47 ± 0.046	0.78 ± 0.146	Synergistic
	CTN 30 µM + DOX 1000 nM	0.29 ± 0.063	0.58 ± 0.122	Synergistic
**DU-145**	CTN 10 µM + DOX 200 nM	0.63 ± 0.033	0.99 ± 0.133	——
	CTN 20 µM + DOX 200 nM	0.42 ± 0.023	0.80 ± 0.035	Synergistic
	CTN 30 µM + DOX 200 nM	0.31 ± 0.036	0.83 ± 0.094	Synergistic
	CTN 10 µM +DOX 1000 nM	0.42 ± 0.031	1.27 ± 0.111	——
	CTN 20 µM + DOX 1000 nM	0.33 ± 0.056	1.14 ± 0.205	——
	CTN 30 µM + DOX 1000 nM	0.26 ± 0.015	1.04 ± 0.035	——

Combination index (CI) among the combinations of two drugs in PC-3 and DU-145 cells were calculated using CompuSyn software. If CI > 1, it denoted antagonism; if CI < 1, it denoted synergism. Data presented as mean ± SD were representative of three independent experiments.
